# Sex Promotes Spatial and Dietary Segregation in a Migratory Shorebird during the Non-Breeding Season

**DOI:** 10.1371/journal.pone.0033811

**Published:** 2012-03-30

**Authors:** Teresa Catry, José A. Alves, Jennifer A. Gill, Tómas G. Gunnarsson, José P. Granadeiro

**Affiliations:** 1 CESAM, Museu Nacional de História Natural, Universidade de Lisboa, Lisboa, Portugal; 2 School of Biological Sciences, University of East Anglia, Norwich, United Kingdom; 3 South Iceland Research Centre, University of Iceland, Hella, Iceland; University of Plymouth, United Kingdom

## Abstract

Several expressions of sexual segregation have been described in animals, especially in those exhibiting conspicuous dimorphism. Outside the breeding season, segregation has been mostly attributed to size or age-mediated dominance or to trophic niche divergence. Regardless of the recognized implications for population dynamics, the ecological causes and consequences of sexual segregation are still poorly understood. We investigate the foraging habits of a shorebird showing reversed sexual dimorphism, the black-tailed godwit *Limosa limosa*, during the winter season, and found extensive segregation between sexes in spatial distribution, microhabitat use and dietary composition. Males and females exhibited high site-fidelity but differed in their distributions at estuary-scale. Male godwits (shorter-billed) foraged more frequently in exposed mudflats than in patches with higher water levels, and consumed more bivalves and gastropods and fewer polychaetes than females. Females tended to be more frequently involved and to win more aggressive interactions than males. However, the number of aggressions recorded was low, suggesting that sexual dominance plays a lesser role in segregation, although its importance cannot be ruled out. Dimorphism in the feeding apparatus has been used to explain sex differences in foraging ecology and behaviour of many avian species, but few studies confirmed that morphologic characteristics drive individual differences within each sex. We found a relationship between resource use and bill size when pooling data from males and females. However, this relationship did not hold for either sex separately, suggesting that differences in foraging habits of godwits are primarily a function of sex, rather than bill size. Hence, the exact mechanisms through which this segregation operates are still unknown. The recorded differences in spatial distribution and resource use might expose male and female to distinct threats, thus affecting population dynamics through differential mortality. Therefore, population models and effective conservation strategies should increasingly take sex-specific requirements into consideration.

## Introduction

Sexual segregation is a widespread feature in several avian taxa and is often linked to sexual dimorphism and to the different roles played by males and females. These differences are mostly noticeable during the breeding period [1]. Nonetheless, sexual segregation outside the breeding season has also been described for a large number of bird species [2]–[4], and two major hypotheses have been postulated to explain this phenomenon. The social dominance hypothesis predicts that the subordinate (smaller, less agressive or otherwise less competitive) individuals are excluded from high quality areas by dominant conspecifics [5]. On the other hand, the specialization hypothesis predicts that segregation arises from niche specialization, either resulting from habitat preferences, differential access to food resources or tolerance to ecological factors [6]–[7].

Among shorebirds, sexual dimorphism is a common trait, frequently involving differences in body size, in which males tend to be smaller than females [8]. Evidence from previous studies suggests that sexual dimorphism in shorebirds has primarily evolved through selection pressures related to breeding processes (e.g. mating systems, parental care or aerial agility; [9]) but ecological processes, such as intraspecific competition for food and differential migration, may have also played an important role [10]–[11]. Among shorebirds, sexual dimorphism in feeding apparatus and specifically in bill length, is more pronounced than in body size, a trait shared with other avian taxa (e.g. grebes, pelicans, woodpeckers). This feature is therefore likely to be of relevance for sex-specific foraging, leading to differences in the spatial distribution of sexes [12]–[13], in diet composition and/or in feeding techniques [14]–[17].

Sex-related spatial segregation can occur at either large or small-scale, resulting in broad geographical differences in the wintering distribution [12], [18], or in local differences in habitat/microhabitat use [4], [19]. Large-scale spatial segregation has been described in detail for the western sandpiper *Calidris mauri*, in which larger-billed females are mostly found in the southern part of the wintering range [12], [20]. Sexual segregation at a small spatial-scale has been more frequently reported in shorebirds. Wintering bar-tailed godwits *Limosa lapponica* in Australia segregate by sex in foraging grounds, with (smaller) males avoiding sand flats which are extensively used by (larger) females [4]. Similarly, male bar-tailed godwits foraging in both European and African intertidal flats rarely feed along the water line whereas females tend to be tide-followers [19], [21]–[22].

Habitat segregation by sex is commonly coupled with dietary segregation, given that particular prey species tend to be associated with specific habitats. For instance, male bar-tailed godwits consume predominantly small bivalves while females prefer worms [19], [21]. As a rule, longer bills provide opportunity for exploiting prey buried deeper and more powerful bills are a pre-requisite to consume hard-shelled prey [15]. Although habitat and diet segregation seem to arise from sexual dimorphism in bill size through differential foraging ability, in most cases it is not clear whether intraspecific competition for food, leading to competitive interactions, also plays a role in the observed foraging behaviour and distribution of sexes in dimorphic shorebird species [4], [21].

Sexual segregation can have important consequences for population dynamics [16]. Individuals exploiting different habitats and resources are expected to experience different pay-offs and ultimately suffer differential mortality promoted by differences in environmental factors such as prey availability and profitability [10], predation risk [23], exposure to parasites [24], pollutants [25], adverse weather events [26] and habitat loss [27]. Regardless of the mechanisms driving sexual segregation, the fact that males and females are spatially or temporally separated or exploit different resources, has implications for management and conservation strategies [28], and should thus be taken into account by both researchers and decision-makers.

The black-tailed godwit *Limosa limosa* is a sexually dimorphic migratory shorebird [29]–[30] with females being ca. 15–18% heavier [31], 6% longer winged and having bills ca. 16% longer than males [29]. European populations of black-tailed godwits breed mainly in the Netherlands (*L.l.limosa*) and Iceland (*L.l. islandica*) and winter across Western Europe, from the United Kingdom and Ireland to the Iberian Peninsula, with the nominate subspecies also present in large numbers in West Africa [32]. Numerous godwits have been sexed and individually colour-ringed across their breeding and wintering areas as part of an international long-term marking program. This provided the rare opportunity to accurately identify individuals of different sexes in the field and investigate differences between male and female godwits in their foraging ecology outside the breeding season. Previous work has suggested that within-estuary sexual segregation in distribution and resource use might occur in this system [32]. However, it is not known whether these differences are primarily a function of sex or body size, neither if sexual segregation arises from niche divergence or dominance-effects. In this study, we used the large number of individually-marked individuals (with known sex and with biometric information) to investigate the extent to which male and female godwits differ in their (a) spatial distribution and site-fidelity, (b) resource use, (c) microhabitat use and (d) interaction behaviour. We also investigate the effect of bill size in resource use among sexes and within birds of the same sex. Based on our findings we explore the consequences of sexual size dimorphism for distribution and resource use and briefly discuss whether the social dominance or specialization hypotheses might have driven sexual segregation in wintering black-tailed godwits.

## Methods

### Study area

This study was carried out at the Tagus estuary, Portugal (38° 45′ N, 09° 50′ W), one of the largest estuaries in Europe and the second most important wetland for waders in Iberia [32]. The Tagus estuary comprises an intertidal area covering about 97 km^2^, mostly composed of mudflats with smaller areas dominated by sandy sediments. Saltmarshes, saltpans and agricultural fields (mainly rice-fields) are also available as feeding and roosting habitats for shorebirds.

Black-tailed godwits from both the *L. l. limosa* and *islandica* subspecies occur in winter on the Tagus estuary. However, there is strong habitat segregation, with Icelandic godwits primarily occurring in intertidal flats whereas continental birds use mainly rice-fields [33]. In the intertidal flats, where this study took place, 2500 to 4000 godwits occur from November to January, ca. 65–75% of which are *islandica* [33].

A total of 224 Black-tailed godwits were captured and individually colour-marked in the Tagus estuary between 2006 and 2010, and these form the majority of the birds used in this study. However, long-term marking of individual Icelandic godwits has been underway since 1993 elsewhere [34]–[35], and some of these birds also winter on the Tagus and were included in the study. The sex of colour-marked birds was determined using a discriminant function analysis of bill and wing measurements (measured to the nearest 0.1 and 1 mm, respectively; [29]). For godwits captured during primary moult (thus missing the wing length measurement), and also for those with discriminant scores close to zero, sexing was carried out by identifying males as those with bill length <88 mm or females with bill lengths >92 mm [29]. The only godwit that did not fit any of these criteria was excluded from further analyses. In order to validate sexing through biometric criteria, twenty nine godwits included in the study were also sexed through molecular techniques. We found 100% match between the two approaches.

**Figure 1:**
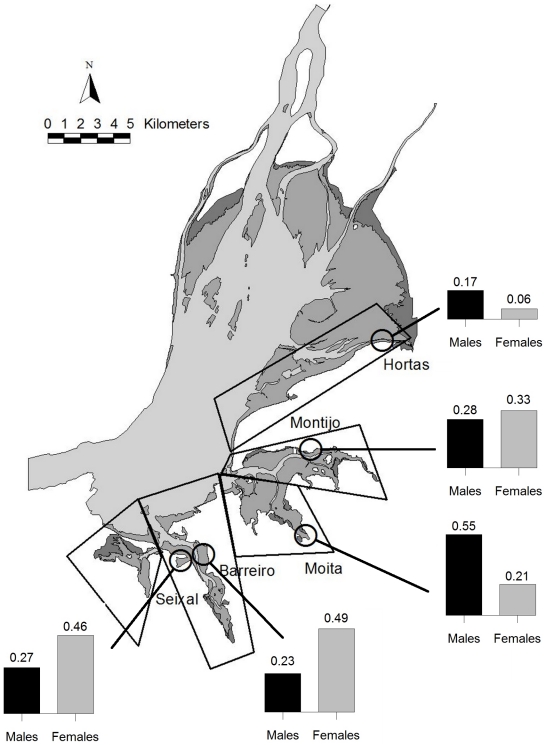
Map of the study areas and distribution of individually colour-marked male (n = 71) and female (n = 33) black-tailed godwits at the Tagus estuary. Grey shading, from dark to light, represents saltmarshes, intertidal flats and immersed areas, respectively. Large polygons in the map represent the location of the areas used to estimate site-fidelity and the circles indicate the five study sites where invertebrate sampling and godwits' foraging observations were carried out. The proportion of male and female godwits in each of the five foraging sites corresponds to the number of birds of each sex observed in each area (during the whole study period) in relation to all birds resighted in all the study areas (because some individuals were recorded in more than one site, the sum of proportions for each sex can be >1). Total numbers of individual male and female (male∶female) godwits recorded: 16∶16 at Barreiro, 12∶2 at Hortas, 39∶7 at Moita, 20∶11 at Montijo and 19∶15 at Seixal.

Except for the site-fidelity analysis (see below), all the work took place at five study sites (Figure 1) located on the southern shore of the estuary, representing important foraging sites in the estuary: Seixal, Barreiro, Moita, Montijo and Hortas (n = 22, 22, 28, 23, and 5 visits, respectively).

### Spatial distribution and site fidelity of godwits

We investigated differences in large-scale spatial distribution between male and female godwits. The estuary was divided into five major areas (Figure 1), where all observations were carried out. These areas encompass one main roost (maximum two) and several adjacent foraging sites, such that a change to a new roost would likely involve a change in foraging site and vice-versa. The whole estuary was surveyed for colour-marked godwits weekly between mid January and mid February and twice a week in March 2010, during both low and high-tide periods. Differences in the spatial distribution of male and female godwits were assessed by comparing the total number of individuals from each sex in the five areas during the whole period. In order to test whether males and females differ in their fidelity to the study areas, we developed the following individual site-fidelity index (SFI):
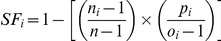
where n*i* is the number of areas used by individual *i, n* is the total number of areas surveyed, *pi* is the observed number of changes between areas performed by individual *i* and *oi* is the total number of observation events of individual *i.* SFI ranges from zero (no site-fidelity) to one (complete site-fidelity). The first term represents the “spatial” component of fidelity (proportion of areas used) and the second one the “temporal” or “behavioural” component (how frequently did birds move among areas). Only individuals observed at least four times during the study period were included in the analysis. This threshold was used in order to avoid including birds with very few observations which could introduce some bias to the estimation of the fidelity index: birds rarely observed could either be less conspicuous individuals whose presence is difficult to record or individuals that did not stay in the estuary for the whole study period. On the other hand, setting the minimum number of observations at a higher level would considerably decrease the number of birds that entered the analysis.

### Resource use

Previous studies in the Tagus estuary have shown that the diet of black-tailed godwits mainly includes the bivalve *Scrobicularia plana*, the polychaete *Hediste diversicolor* and the gastropod *Hydrobia ulvae* [36]. Thus, prey abundance of these three prey items was estimated at the five study sites (Figure 1) in early March 2010 (assuming that those are representative from the winter period [31]), by taking 10 sediment cores (86.6 cm^2^, 20 cm deep) at randomly located points within the area used by foraging godwits. Sediment cores were immediately sieved using a 1-mm mesh size. All invertebrates were preserved in 70% ethanol, taken to a laboratory and later identified and counted. The difference in mean culmen length between male and female godwits is ca. 1.5 cm, and therefore obtaining separate estimates of availability for males and females would be logistically very challenging. Given that invertebrate prey are mostly concentrated in the upper 0–8 cm of the sediment [37]–[38], we considered that the availability of prey was similar for both sexes.

Data on the feeding behaviour and diet of godwits were collected at the five study sites (Figure 1) between November and March, during the winters of 2009–2010 and 2010–2011 (Hortas was surveyed only in the first winter). Individually-marked godwits were observed while foraging with a ×20–60 zoom telescope during five consecutive periods of 30 seconds, in which the identity (*S. plana, H. diversicolor and H. ulvae*), number, and size of each prey item consumed were recorded. If a bird flew or stopped foraging before at least four 30-sec observations had been completed, the observations were discarded. Prey identity was assessed visually by its shape and by the conspicuous differences in foraging behaviour for different prey: deep probes when preying on polychaetes (>30% of bill length is inserted), continuous shallow pecks when preying on bivalves (<20% of bill length is inserted), and stitching on the surface of the sediment when preying on gastropods ([31], pers. obs). Sizes of *S. plana* and *H. diversicolor* were estimated visually in relation to bill length and classified as small, medium, large or very-large. Average prey length of each class was reconstructed from both prey fragments found in faeces and prey sampling carried out in a previous study at the same sites (*S.plana*: small: 3–5.5 mm; medium: 5.6–9.5 mm; large: 9.6–14.5 mm; very large: 14.6–20.0 mm; *H. diversicolor*: small: 3–9.9 mm; medium: 10–19.9 mm; large: 20.0–49.9 mm; very large: >50 mm, [39]). An index of average prey size consumed was calculated for these two invertebrates by multiplying the number of prey consumed by a factor of 1 to 4, corresponding to increasing prey size class (small, medium, large and very large, respectively). Mean size of *H.ulvae* varied little and was defined as 4 mm [39]. We calculated ash-free dry mass (AFDM) of each size-class of prey using species-specific equations published for the Tagus estuary ([36] for *S. plana* and *H. diversicolor*; [40] for *H. ulvae*). The biomass (mg) of each prey type consumed by an individual was then averaged over the five consecutive 30-second observations and dietary composition was calculated as the proportions of each prey type. Repeated observations of the same individual foraging on different days or two blocks of five observations on the same day separated by >45 minutes were considered to represent different (yet not entirely independent, see statistical analysis) events and, accordingly, distinct estimates of dietary composition were calculated for each individual during each separate observation event.

Prey selection by male and female godwits was calculated using the Manly's preference index (α), assuming constant prey populations in both study seasons:
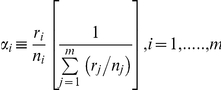
where *α_i_* is the preference for prey *i* when *m* prey types are available, *r_i_* is the proportion of the prey *i* in the diet and *n_i_* is the proportion of the prey *i* in the environment [41]. Values of *α_i_*>1/m indicate positive selection, values of *α_i_*<1/m indicate prey avoidance and values of *α_i_* close to 1/m suggest that prey type is consumed in the same proportions as it is available in the environment.

### Microhabitat use

For each 30-sec observation we also classified microhabitat use regarding water level of foraging areas into three classes: (1) no water – bird feeding on exposed mudflats or oyster beds, (2) water below knee and (3) water above knee.

### Interaction behaviour

In 2010–2011, during the foraging observations of godwits, all aggressive interactions (defined as displacement trials with or without physical contact) between the focal bird and any nearby godwit were recorded and, whenever possible, we also noted whether the focal bird was the “winner” of the interaction.

### Statistical analyses

Differences in the spatial distribution of male and female black-tailed godwits in the Tagus estuary were assessed by comparing, with a chi-squared test, the number of individuals of each sex observed in each of the five study areas throughout the whole study period. To investigate differences in the individual site-fidelity (SFI) between male and female godwits we used a t-test.

The relationship between dietary composition (measured as the proportional contribution to diet of each prey type, expressed in terms of biomass consumed), and the sex and culmen length of individual godwits was investigated using Generalized Linear Mixed Model (GLMM). fitted by maximizing restricted log-likelihood [42]. One model was built for each one of the three prey types consumed. Prey density in the sediment (number of prey items/m^2^, obtained from the invertebrate sampling) was included as a control variable, given that the consumption of a particular prey is likely to be linked with its availability. We also included in the models the variable “date”, defined as the number of days elapsed since the first of October (each year), to control for potential variation in consumption of each prey during the course of both winters. These models used a Binomial error distribution with a logit link function.

GLMMs were also used to test the effect of godwit sex and culmen length on the average size of *S. plana* and *H.diversicolor* consumed (using the average prey size index), and again prey density and date were set as covariates. For prey size, the models used a Gaussian error distribution with an identity link function.

In all GLMMs, the inclusion of both sex and culmen length enabled us to test the relationships within each particular sex. Given that most of the data obtained on foraging godwits consists of repeated observations over the same individuals, bird identity was included as a random factor. Models were initially fitted with all predictors and their interactions and then compared with increasingly simpler nested models with similar random structure, constructed by deletion of the non-significant fixed term until only significant terms (p<0.05) remained in the model. Comparisons between models were based on log-likelihood ratio tests [43].

Microhabitat use by male and female godwits was compared using compositional analysis [44]. Given that the individual frequencies of occurrence in different habitat types (no water, water below knee and water above knee) always sum to 1 and are not inter-independent (unit-sum constraint), we used an isometric log-ratio transformation, which converts proportions into real coordinates, while preserving relevant metric properties. These transformed data were then entered in a Multivariate Analysis of Variance (MANOVA), with sex as a fixed factor.

In order to compare the frequency of occurrence of agonistic interactions between male and female godwits and the proportion of interactions won by each sex-class, GLMMs were fitted using a binomial error distribution and a logit link function, with individual-identity as random factor.

All analyses were carried out with R v.2.11.1 (R Development Core Team, 2010) using packages “nlme” [45] and “compositions” [46]. Means are presented ± SE, except if otherwise stated.

## Results

### Spatial distribution and site fidelity of godwits

In total, 104 individually colour-marked black-tailed godwits were identified during the study period foraging at the five selected study sites, 71 of which were males and 33 were females. These male and female godwits showed an unequal distribution across the five study foraging areas of the Tagus estuary (χ^2^_4_ = 15.07, p<0.01; Figure 1). The level of individual fidelity to the five foraging sites was high and very similar between male and female godwits (SFI = 0.93±0.09 n = 61 and SFI = 0.91±0.17 n = 27, respectively; t_86_ = −0.622, p = 0.536). This result was not influenced by resighting probabilities (calculated as the ratio between the number of resightings of individual godwits and the total number of visits) given that this parameter was not significantly different between sexes (males = 0.51±0.03; females = 0.57±0.04; Wilcoxon Test W = 2181.5, p = 0.220).

### Resource use

Densities of *H. diversicolor*, *S. plana* and *H. ulvae* varied significantly among the five study sites (Kruskal-Wallis χ^2^_4_ = 20.86 p<0.001, χ^2^_4_ = 16.81 p<0.01 and χ^2^_4_ = 20.30 p<0.001, respectively; Table 1).

**Table 1:** Densities of *Scrobicularia plana*, *Hediste diversicolor* and *Hydrobia ulvae* and sex-specific prey selection (α) by black-tailed godwits at the five study sites of the Tagus estuary.

	*Scrobicularia plana*			*Hediste diversicolor*			*Hydrobia ulvae*		
	density	α females	α males	density	α females	α males	density	α females	α males
Barreiro	473.4±152.5	**0.425**	**0.865**	1547.3±326.7	**0.542**	0.111	2736.7±742.0	0.032	0.024
Seixal	196.3±75.1	**0.504**	**0.833**	369.5±147.9	**0.496**	0.140	4642.0±750.7	0	0.027
Montijo	692.8±211.5	**0.450**	**0.805**	288.7±96.2	**0.550**	0.180	8625.9±2528.0	0.0003	0.014
Moita	57.7±57.7	**0.767**	**0.888**	219.4±91.8	0.218	0.095	5046.2±1405.9	0.015	0.017
Hortas	69.3±35.3	**1**	**1**	103.9±36.3	0	0	681.3±178.4	0	0.0004

Invertebrate density is expressed as mean ± SE, in ind.m^−2^. Prey selection was estimated using the Manly's preference index (α). Values of α in bold indicate positive selection of a specific prey (see methods).

**Figure 2:**
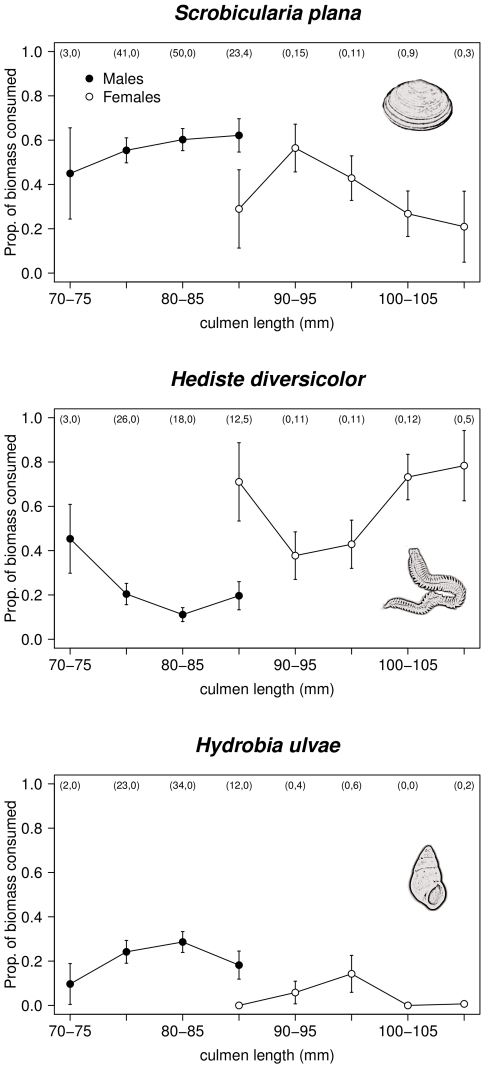
Dietary composition (mean contribution of each prey to the total biomass consumed ± SE) of male and female black-tailed godwits in relation to culmen length (mm). Number of observations of male and female godwits, respectively, in each culmen size-class is presented in bracket in the top of each figure.

The proportion of *S. plana* and *H. diversicolor* consumed by godwits increased significantly with prey density (GLMM: t_368_ = 4.568, p<0.001 and t_368_ = 3.674, p<0.001, respectively; Table 2). However, the proportion of *H. ulvae* in the diet was not influenced by its abundance (Table 2). Male godwits consumed more *H. ulvae* (GLMM: t_151_ = 3.884, p<0.001; Table 2) and fewer *H. diversicolor* (GLMM: t_151_ = −6.582, p<0.001; Table 2) than females, and the consumption of these prey was not influenced by culmen length in either males or females (Table 2 and Figure 2). Overall, males also consumed more *S.plana* than females (Figure 2), but the proportion of these bivalves in the diet was differently affected by culmen length within the two sexes (GLMM: t_151_ = 2.171, p = 0.032; Table 2). Longer-billed males tend to consume more *S. plana* than short-billed males, whereas the reverse was true for females (Table 2 and Figure 2). Only the consumption of *H.ulvae* was affected by date, with godwits preying more upon this prey as the winter season progressed.

**Table 2:** Generalized linear mixed models relating dietary composition of black-tailed godwits to prey density, culmen length, sex and date.

*Scrobicularia plana*					
Model selection	df	AICc	Test	L. ratio	p-value
1 Prey density+Culmen*Sex+Date	7	538.3			
2 Prey density+Culmen*Sex	6	535.6	2 vs 1	0.006	0.936
3 Prey density+Culmen+Sex	5	538.6	3 vs 2	5.618	0.018

Final model	estimate	SE	z-value	p-value
Intercept	2.6390	1.1638	2.267	0.024
Prey density	0.0007	0.0001	4.568	0.000
Culmen	−0.0238	0.0119	−1.995	0.048
Sex (Males = 1, Females = 0)	−3.0480	1.5450	−1.973	0.050
Culmen:Sex (Males = 1, Females = 0)	0.0374	0.0173	2.171	0.032

*Hediste diversicolor*					
Model selection	df	AICc	Test	L. ratio	p-value
1 Prey density+Culmen*Sex+Date	7	373.4			
2 Prey density+Culmen*Sex	6	372.4	2 vs 1	0.063	0.802
3 Prey density+Culmen+Sex	5	371.9	3 vs 2	0.553	0.457
4 *Prey density+Sex*	4	371.6	4 vs 3	0.816	0.366
5 Prey density	3	403.5	5 vs 4	32.815	<0.001
6 Sex	3	383.9	6 vs 4	13.244	<0.001

Final model	estimate	SE	z-value	p-value
Intercept	−0.4680	0.3665	−1.277	0.202
Prey density	0.0010	0.0003	3.827	<0.001
Sex (Males = 1, Females = 0)	−2.2665	0.3768	−6.016	<0.001

*Hydrobia ulvae*					
Model selection	df	AICc	Test	L. ratio	p-value
1 Prey density+Culmen*Sex+Date	7	395.0			
2 Prey density+Culmen+Sex+Date	6	394.0	2 vs 1	0.079	0.778
3 Culmen+Sex+Date	5	382.4	3 vs 2	0.000	1.000
4 *Sex+Date*	4	381.5	4 vs 3	0.271	0.603
5 Sex	3	387.6	5 vs 4	7.034	0.008
6 Date	3	397.9	6 vs 4	17.452	<0.001

Final model	estimate	SE	z-value	p-value
Intercept	−5.4679	0.8790	−6.220	<0.001
Sex (Males = 1, Females = 0)	2.2946	0.6962	3.296	<0.001
Date	0.0141	0.0052	2.693	0.007

Dietary composition is expressed as proportion of each prey type consumed in biomass. The table presents comparisons of increasingly simpler nested models, using likelihood-ratio tests (L.ratio) and coefficients of best significant models, fitted by Restricted Maximum Likelihood. Bird identity was treated as a random factor and “*” stands for interaction between variables. The best model is presented in italics and the estimation of the coefficients (estimate) is given with standard errors (SE). AIC values corrected for finite sample sizes (AICc) are presented for comparative purposes only. t-tests and the corresponding p-values are used to test the significance of each term of the final model.

In order to test for the potential effects of inter-annual variation in invertebrate prey, models were re-run with data on invertebrate densities obtained during 2007 (involving two sites and two species: *S. plana* and *H. diversicolor*, [31]). With the exception of an interaction term between culmen and sex in *S.plana* model, the same variables were retained in the models (Table S1). Moreover, the way in which they influenced the consumption (increasing or decreasing) was also unchanged.

Both females and males positively selected *S. plana* at all study areas, but only females showed active selection for *H. diversicolor* (Table 1). Female selection for *H. diversicolor* also occurred irrespective of which prey type was the most abundant at specific sites (Table 1).

**Figure 3:**
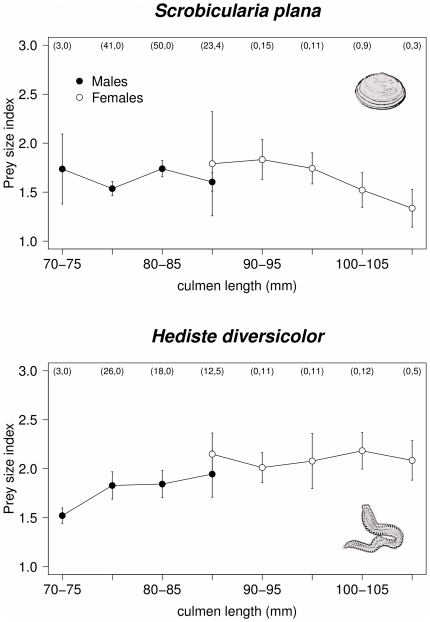
Size of prey (prey size index ± SE) consumed by male and female black-tailed godwits in relation to culmen length (mm). Number of observations of male and female godwits, respectively, in each culmen size-class is presented in bracket in the top of each figure. The index of prey size was calculated by multiplying the number of prey consumed by a factor of 1 to 4, corresponding to increasing prey size class (small, medium, large and very large, respectively), and dividing this value by the number of consumed prey.

Male and female godwits also showed some differences in the size of consumed prey: females exploited larger *H. diversicolor* than males (GLMM: t_83_ = −3.017, p = 0.003; Table 2), but there were no differences in size of *S. plana* consumed (Table 3). Within each sex, culmen length did not affect the size of prey captured (Table 3 and Figure 3). Godwits tended to consume larger bivalves later in the season (Table 3).

**Table 3:** Generalized linear mixed models relating size of prey consumed by black-tailed godwits with prey density, culmen length, sex and date.

*Scrobicularia plana*					
Model selection	df	AICc	Test	L. ratio	p-value
1 Prey density+Culmen*Sex+Date	8	2542.2			
2 Prey density+Culmen+Sex+Date	7	2542.1	2 vs 1	1.031	0.310
3 Prey density+Sex+Date	6	2541.1	3 vs 2	0.0003	0.986
4 Sex+Date	5	2540.2	4 vs 3	0.029	0.865
5 *Date*	4	2540.8	5 vs 4	1.663	0.197
6 Null model	3	2557.8	6 vs 5	18.018	0.000

Final model	df	estimate	SE	t-value	p-value
Intercept	1128	1.2682	0.100	12.652	0.000
Date	1128	0.0035	0.001	4.289	0.000

*Hediste diversicolor*					
Model selection	df	AICc	Test	L. ratio	p-value
1 Prey density+Culmen*Sex+Date	8	1619.3			
2 Prey density+Culmen+Sex+Date	7	1618.4	2 vs 1	0.096	0.757
3 Prey density+Sex+Date	6	1617.6	3 vs 2	0.170	0.680
4 Sex+Date	5	1616.8	4 vs 3	0.271	0.603
5 *Sex*	4	1618.4	5 vs 4	2.613	0.106
6 Null model	3	1625.6	6 vs 5	8.224	0.004

Final model	df	estimate	SE	t-value	p-value
Intercept	550	2.0591	0.081	25.365	0.000
Sex (Males = 1, Females = 0)	83	−0.3276	0.109	−3.017	0.003

The table presents comparisons of increasingly simpler nested models, using likelihood-ratio tests (L.ratio) and coefficients of best significant models, fitted by Restricted Maximum Likelihood. Bird identity was treated as a random factor and “*” stands for interaction between variables. The best model is presented in italics and the estimation of the coefficients (estimate) is given with standard errors (SE). AIC values corrected for finite sample sizes (AICc) are presented for comparative purposes only (differences in AICc values lower than 2 between candidate models were not valorised; [59]). t-tests and the respective p-values are used to test the significance of each tern of the final model.

### Microhabitat use

**Figure 4:**
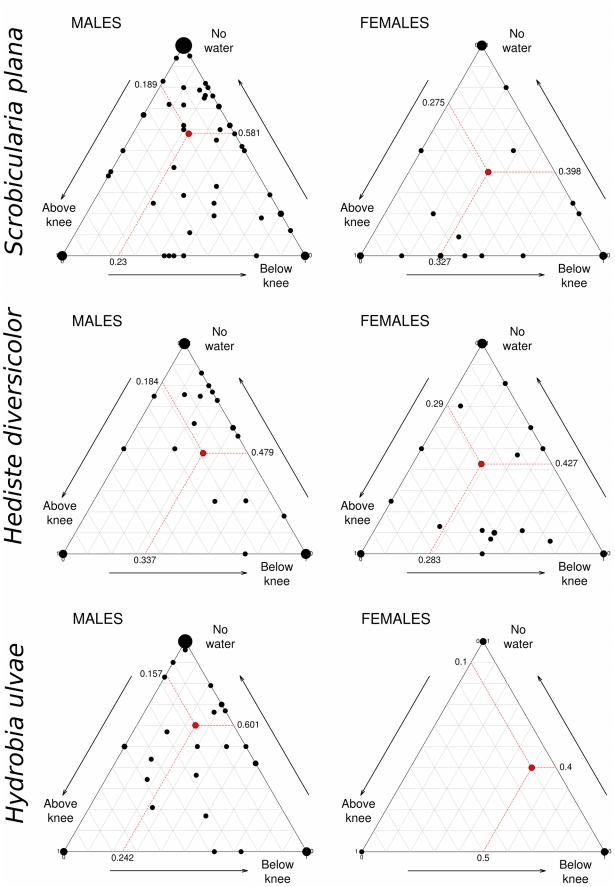
Microhabitat use by male and female godwits in the Tagus estuary when feeding upon *Scrobicularia plana, Hediste diversicolor* and *Hydrobia ulvae*. Microhabitat was classified into three categories according to the water level of foraging areas: (1) no water – birds feeding on exposed mudflats or oyster beds, (2) water below knee and (3) water above knee. Black dots in the ternary diagrams represent the proportions of microhabitat use calculated for each individual (in each study-season). Dot size (area) is proportional to the number of individual observations with the same coordinates. Average values of microhabitat use for each prey type and sex-class are represented by red dots; red-lines guide the readings in ternary graphics. Arrows outside the diagram indicate the increasing use of each specific microhabitat class.

While foraging exclusively upon *S. plana* and *H. diversicolor*, male and female godwits showed significant differences in microhabitat use (MANOVA: Pillai-Bartlett statistic = 0.049, F_2,129_ = 3.295, p = 0.040 and Pillai-Bartlett statistic = 0.114, F_2,80_ = 3.295, p = 0.008, respectively), but no differences were found when birds fed on *H. ulvae* (Pillai-Bartlett statistic = 0.015, F_2,68_ = 0.519, p = 0.597). Males foraging upon *S.plana* and *H.diversicolor* tended to occur more frequently in exposed mudflats, whereas females occurred more frequently in patches with high water levels (Figure 4).

### Interaction behaviour

Aggressive interaction between focal godwits and other foraging birds occurred in ca. 3.5% of all observations (n = 2086). At Barreiro, where the ratio male:female was balanced (16∶16), aggressive interactions were also rarely observed (4.9%, n = 571). Overall, agonistic behaviour was more predominant in focal females (GLMM: z = −2.453, p<0.05), and females won the aggression interactions more often than males (GLMM: z = −2.431, p<0.05).

## Discussion

Sexual dimorphism is widespread among migratory shorebirds [16], and yet relatively few studies have investigated the consequences of sex-related morphological differences in the foraging decisions of these species during the non-breeding season. On the other hand, most studies suggesting that segregation between sexes is directly driven by sexual dimorphism failed to investigate in detail whether morphological differences in individuals of the same sex also promoted different ecological responses. This study provides compelling evidence of spatial segregation of male and female wintering black-tailed godwits at both estuary and microhabitat scales, as well as sexual dietary segregation. Although dimorphism in bill size seemed the likely a-priori explanation for the recorded sexual segregation, the relationship between bill length and resource use did not hold for either sex separately. The only exception was the model for *S. plana* consumption, which indicates a slight, yet significant, interaction between sex and culmen length. According to this model, while females decreased (slightly) the consumption of *S. plana* with increasing culmen length, males showed the opposite relationship. This discrepancy, together with the evidence coming from the other prey species, support to the idea that differences in the foraging habits of godwits are primarily a function of sex, rather than bill size. Hence, the exact mechanisms through which this segregation operates remain still poorly know.

### Sexual segregation in the spatial distribution, resource use and microhabitat use of wintering black-tailed godwits

Despite the lack of a clear pattern of sexual segregation across their wintering range and of a balanced sex-ratio at the Tagus estuary [31], male and female black-tailed godwits showed a clear spatial segregation at the estuary scale. Study sites did not represent *a priori* distinct habitat types in terms of their apparent physical characteristics. To some extent, this observation supports the idea that the disparate distribution of godwits does not seem to be driven by sex-specific preferences for particular features of the habitat. However, and despite the lack of obvious environmental differences between sites, prey density differed significantly, supporting the idea of unequal quality of the areas as foraging grounds. Site quality or suitability might, however, meet different criteria for different intra-specific groups (e.g. sex, age, etc), especially if these groups differ in their energetic requirements or foraging skills and, consequently, in their diet preferences [10]. For example, in Guinea-Bissau, whimbrels *Numenius phaeopus* are sexually segregated in their foraging areas, with longer-billed females being more abundant in intertidal flats where fiddler crabs *Uca tangeri* have higher densities [19]. Profitable fiddler crabs are buried beyond the reach of shorter-billed males which forage preferentially in areas along the water line, where alternative prey occurs [19]. Therefore, the quality of foraging sites cannot be evaluated equally for male and female whimbrels, but rather considering the availability of the preferred prey for each sex.

Male and female black-tailed godwits at the Tagus estuary showed significantly different diets, with males consuming comparatively more bivalves and gastropods and fewer polychaetes than females. Despite the positive correlation between density and consumption of both *S. plana* and *H. diversicolor*, and the differences in prey density among sites, the differential distribution of male and female godwits cannot be the sole explanation for such dietary divergences. Although there is an apparent opportunistic feeding behaviour, as godwits increase the consumption of a particular prey where it is more abundant (except for *H. ulvae*), the magnitude of this response is not equal for male and females, suggesting some level of intra-sex specialization. This is clearly supported by data on prey selection: unlike females that positively select *S. plana* (at all sites) and *H. diversicolor* (where this prey has higher densities), male godwits exclusively select *S. plana*. These results provide some clues to classify and rank the quality of study areas as a function of bivalve and polychaete density, and suggest that females tend to avoid areas with low abundance of these particular prey. However, it should be stressed that apart from prey abundance, many other factors might contribute to the overall quality of foraging areas and, for instance, high levels of intra and inter-specific competition, interference and predation risk are known to depress intake rate [22], [47].

Sex-related differences in the diet composition of shorebirds have been most frequently linked with sexual dimorphism in their feeding apparatus, namely in bill length and shape [15], [17], [19], [21]. Bill length is particularly important for birds that prey upon burying invertebrates, as longer bills provide access to deeper layers of the sediment [20]. In the Tagus estuary, the increased occurrence of female black-tailed godwits in sites with higher densities of *H. diversicolor*, as well as the higher consumption of this polychaete in relation to males, is likely linked to the accessibility of this type of prey. Burrowing depth of *H. diversicolor* increases with body size [37], and larger classes of polychaetes are thus less accessible to shorter-billed males. Moreover, a previous study in the Tagus estuary showed that the estimated profitability of *S. plana* is higher than *H. diversicolor* for small, medium and large prey sizes, and only very large *H. diversicolor*, the ones more deeply-buried, are more profitable then *S. plana* of the same size class [31]. Thus, consuming polychaetes seems to be more profitable for females, which not only consumed more *H. diversicolor* than males but also preyed upon larger items. On the other hand, all size classes of *S. plana* consumed by godwits are accessible, as burrowing depth for this prey does not exceed the length of the shortest godwit bill [48]. This is in accordance with the lack of differences in the size of bivalves taken by males and females at the Tagus estuary.

Distinct prey choice frequently leads to sexual segregation in habitat or microhabitat use, which is mainly due to prey distribution or accessibility [4]. Sexual microhabitat segregation was also evident in this study, with female black-tailed godwits feeding frequently in patches with water, while males concentrated on exposed flats. This could be expected for birds preying upon polychaetes, which are known to be more accessible in submerged mudflats [19], but was also evident for *S. plana*. Our results are in line with observations made in bar-tailed godwits, which show that female consume more polychaetes and forage in deeper water than males [17], [19], [21].

### Drivers of sexual segregation in foraging black-tailed godwits: social dominance or morphological specialization?

The social dominance hypothesis builds on the idea of exclusion of subordinate individuals by their dominant conspecifics [5]. Patterns of segregation due to social dominance have been previously described for shorebirds and can include differences in the spatial distribution or habitat use of foraging birds [23], [49] or differences in foraging techniques [50]. To the best of our knowledge, spatial segregation (excluding small-scale microhabitat segregation) in shorebirds as a result of social dominance was only reported as an age-related (rather then sex-related) process: juvenile oystercatchers *Haematopus ostralegus* and redshanks *Tringa totanus* are displaced to sub-optimal habitats by adult conspecifics [23], [49]. Nonetheless, habitat segregation as a result of sex-related dominance has been described for other bird groups, such as raptors [51] and passerines [3]. Female black-tailed godwits are considerably larger than males, and are thus the presumed dominant sex. Our results support this idea as we found females to be more frequently involved and to win the majority of aggressive interactions between foraging godwits. Nonetheless, the recorded number of agonistic events was rather low (even in study sites with balanced sex ratio), providing only a weak evidence to support the social dominance hypothesis as a driver of the observed spatial segregation.

An alternative hypothesis to explain sexual segregation is that niche specialization is promoted by distinct habitat and/or diet preferences, as a result of morphological differences between the sexes [2], [6]. In black-tailed godwits, females (the larger sex) should experience higher food demands [16] and, despite also having slightly higher energy assimilation efficiency [52], might still need to forage upon more energetic, on larger prey and/or for longer periods than males. Secondly, longer-billed females can access deep-buried prey items that will often be unavailable to males. Different preferences or skills of male and females might contribute to reduce intra-specific competition [1]. However, the morphological specialization hypothesis also seems to fail in providing a compelling explanation for diet segregation. If the differences in the consumption of polychaetes are related to prey accessibility and thus conditioned by bill length, we would expect to find a significant positive relationship between bill length and the consumption of *H. diversicolor* in both males and females. Indeed, regardless of gender, longer billed individuals have access to deeper levels in the sediment and thus can potentially reach deeper buried polychaetes. The absence of this relationship could potentially be explained by a threshold value in the bill length of godwits above which the regular exploitation of *H. diversicolor* becomes profitable [31], assuming that this value is well within the bill size range of females but mainly outside the range of males.

The social dominance and the specialization hypotheses frequently assume similar causes and predict analogous consequences: both might arise from sexual dimorphism in body size and both might promote trophic niche divergence [6], [53]. In addition, the two mechanisms are not mutually exclusive and can co-occur and interact in complex ways [6], making the separation of causes and effects extremely difficult. For example, in oystercatchers, while age-segregation in foraging habits result mainly from social dominance, sexual segregation is related to foraging specialization, arising from differences in bill morphology [10], [49]. In this study, the observed trophic niche divergence between male and female godwits might have resulted as a consequence of both dominance and specialization hypotheses.

### Sexual segregation: implications for conservation

Sexual segregation may have considerable impact on population dynamics of birds, mostly through differential mortality [6], [16], [54]. Although sex-biased mortalities have been reported in several shorebird species [10], [55]–[56] we only took notice of one study that presented evidence for differential mortality being driven by sexual segregation in resource and habitat use [10]. In the Exe estuary, female oystercatchers that feed upon worms and clams have lower intake rates and lower body condition than mussel-eater males [10]. Moreover, females frequently forage in fields, suffering higher predation risk and exposure to parasites [16], all these factors leading to a higher mortality.

At the Tagus estuary, male and female black-tailed godwits have different probabilities of occurrence in each site, thus site-specific risks might affect populations of each sex differently. Given the strong connection between roosting and foraging sites [57], the loss of a particular roost (e.g. as a result of land reclamation [58]) might have unequal impacts in the populations of male and female godwits. On the other hand, declines or increased variability in the stock of a particular prey are likely to primarily affect the sex-class that more strongly depends on such resource. For example, reduction of untreated wastewaters discharges in the Tagus estuary are likely to affect the abundance of *H. diversicolor* near wastewater outfalls [39], thus potentially affecting female godwits disproportionally.

The spatial and temporal segregation of males and females within a population should therefore be taken into account by both researchers and managers. Up-to-date knowledge on the population dynamics of one species, considering sex-specific parameters, is of high relevance to assess its health status and to further delineate conservation programmes.

## Supporting Information

Table S1 **Generalized linear mixed models relating dietary composition of black-tailed godwits to prey density (including invertebrate density data from 2007), culmen length, sex and date.** Dietary composition is expressed as proportion of each prey type consumed in biomass. The table presents comparisons of increasingly simpler nested models, using likelihood-ratio tests (L.ratio) and coefficients of best significant models, fitted by Restricted Maximum Likelihood. Bird identity was treated as a random factor and “*” stands for interaction between variables. The best model is presented in italics and the estimation of the coefficients (estimate) is given with standard errors (SE). AIC values corrected for finite sample sizes (AICc) are presented for comparative purposes only. t-tests and the corresponding p-values are used to test the significance of each term of the final model. The variable “Prey density” includes data from 2007 (two sites – Barreiro and Hortas; [31]).
(DOC)
