# Supplementary material for: Sex Promotes Spatial and Dietary Segregation in a Migratory Shorebird during the Non-Breeding Season
Source: PLoS One. 2012 Mar 30;7(3):e33811. doi: 10.1371/journal.pone.0033811 (PMC3316497; doi:10.1371/journal.pone.0033811)
Supplement: Table S1 — Generalized linear mixed models relating dietary composition of black-tailed godwits to prey density (including invertebrate density data from 2007), culmen length, sex and date. Dietary composition is expressed as proportion of each prey type consumed in biomass. The table presents comparisons of increasingly simpler nested models, using likelihood-ratio tests (L.ratio) and coefficients of best significant models, fitted by Restricted Maximum Likelihood. Bird identity was treated as a random factor and “*” stands for interaction between variables. The best model is presented in italics and the estimation of the coefficients (estimate) is given with standard errors (SE). AIC values corrected for finite sample sizes (AICc) are presented for comparative purposes only. t-tests and the corresponding p-values are used to test the significance of each term of the final model. The variable “Prey density” includes data from 2007 (two sites – Barreiro and Hortas; [31]). (DOC) [file pone.0033811.s001.doc]

**Table S1. Generalized linear mixed models relating dietary composition of black-tailed godwits to prey density (including invertebrate density data from 2007), culmen length, sex and date.**

| ***Scrobicularia plana*** | | | | | |
| --- | --- | --- | --- | --- | --- |
| **Model selection** | **df** | **AICc** | **Test** | **L. ratio** | **p-value** |
| 1 Prey density+Culmen*Sex+Date | 7 | 537.5 |  |  |  |
| *2* Prey density+Culmen*Sex | 6 | 535.7 | 2 vs 1 | 0.208 | 0.649 |
| 3 Prey density+Culmen+Sex | 5 | 537.3 | 3 vs 2 | 3.534 | 0.060 |
| 4 *Prey density+Sex* | 4 | 535.5 | 4 vs 3 | 0.236 | 0.627 |
| 5 Prey density | 3 | 549.9 | 5 vs 4 | 16.385 | <0.001 |
|  |  |  |  |  |  |
| **Final model** |  | **estimate** | **SE** | **z-value** | **p-value** |
| Intercept |  | -1.3240 | 0.2435 | -5.438 | <0.001 |
| Prey density |  | 0.0028 | 0.0005 | 5.055 | <0.001 |
| Sex (Males=1, Females=0) |  | 0.9950 | 1.2410 | 4.129 | <0.001 |
|  |  |  |  |  |  |
| ***Hediste diversicolor*** | | | | | |
| **Model selection** | **df** | **AICc** | **Test** | **L. ratio** | **p-value** |
| 1 Prey density+Culmen*Sex+Date | 7 | 380.8 |  |  |  |
| 2 Prey density+Culmen*Sex | 6 | 378.9 | 2 vs 1 | 0.091 | 0.763 |
| 3 Prey density+Culmen+Sex | 5 | 377.5 | 3 vs 2 | 0.599 | 0.439 |
| 4 *Prey density+Sex* | 4 | 376.3 | 4 vs 3 | 0.816 | 0.366 |
| 5 Prey density | 3 | 407.7 | 5 vs 4 | 33.428 | <0.001 |
| 6 Sex | 3 | 386.9 | 6 vs 4 | 12.592 | <0.001 |
|  |  |  |  |  |  |
| **Final model** |  | **estimate** | **SE** | **z-value** | **p-value** |
| Intercept |  | -0.3295 | 0.3484 | -0.946 | 0.202 |
| Prey density |  | 0.0006 | 0.0002 | 3.740 | <0.001 |
| Sex (Males=1, Females=0) |  | -2.2847 | 0.3764 | -6.069 | <0.001 |

Dietary composition is expressed as proportion of each prey type consumed in biomass. The table presents comparisons of increasingly simpler nested models, using likelihood-ratio tests (L.ratio) and coefficients of best significant models, fitted by Restricted Maximum Likelihood. Bird identity was treated as a random factor and “*” stands for interaction between variables. The best model is presented in italics and the estimation of the coefficients (estimate) is given with standard errors (SE). AIC values corrected for finite sample sizes (AICc) are presented for comparative purposes only. t-tests and the corresponding p-values are used to test the significance of each term of the final model. The variable “Prey density” includes data from 2007 (two sites – Barreiro and Hortas; [31]).
